# Quantifying morphologic variations as an alternate to standard response criteria for unresectable primary liver tumors after checkpoint inhibition therapy

**DOI:** 10.1007/s11547-024-01937-1

**Published:** 2024-12-10

**Authors:** Laetitia Saccenti, Nicole Varble, Tabea Borde, Andrew S. Mikhail, Michael Kassin, Elliot Levy, Sheng Xu, Lindsey A. Hazen, Ifechi Ukeh, Cyndi Vasco, Austin G. Duffy, Changqing Xie, Cecilia Monge, Donna Mabry, Tim F. Greten, Bradford J. Wood

**Affiliations:** 1 Center for Interventional Oncology, Radiology and Imaging Sciences, Clinical Center, National Institutes of Health, 10 Center Drive, Bethesda, MD 20892 USA; 2 Henri Mondor’s Institute of Biomedical Research - Inserm, U955 Team No. 18, Créteil, France; 3 Philips Research of North America, Cambridge, MA USA; 4 Gastrointestinal Malignancies Section, Thoracic and GI Malignancies Branch, Center for Cancer, Research, National Cancer Institute, National Institutes of Health, Bethesda, MD 20892 USA

**Keywords:** Immunotherapy, Follow-up studies, Response evaluation criteria in solid tumors, Liver neoplasms, Retrospective studies

## Abstract

**Purpose:**

The aim of this study was to assess the feasibility of quantifying morphologic changes in tumors during immunotherapy, as a reflection of response or survival.

**Methods and materials:**

A retrospective single-center analysis was performed in patients with unresectable liver cancer previously enrolled in clinical trials combining immunotherapy (tremelimumab ± durvalumab) and locoregional treatment (either ablation or transarterial chemoembolization). Conventional response (RECIST 1.1) was assessed at 6-month follow-up. For morphologic assessment, the largest target lesion was manually segmented on axial slices in two dimensions using contrast-enhanced CT. Solidity and circularity of tumors were calculated at baseline, 3-month follow-up, and at 6-months follow-up. Survival analysis was performed.

**Results:**

From the 68 patients enrolled in clinical trials, 28 did not have target lesions separate from lesions treated by locoregional therapies, and 3 had no follow-up imaging. Thirty-seven patients (9 with biliary cancer and 28 with hepatocellular carcinoma) were included. Shape features and shape variation were not correlated with RECIST 1.1 status at 6-month follow-up. However, patients with low solidity tumors at 6-month follow-up showed poorer prognosis compared with patients with high solidity tumors at 6-month follow-up (*p* = 0.01). Solidity variation analysis confirmed that a decrease of tumor solidity at 6-month follow-up was associated with poorer prognosis (*p* = 0.01). No association was found between shape features at baseline or shape features at 3-month follow-up with overall survival.

**Conclusion:**

Evolution and variation of tumor morphology during treatment may reflect or correlate with outcomes and contribute toward adapted response criteria.

## Introduction

The therapeutic landscape for patients with advanced liver cancer is rapidly evolving. More than 200 clinical trials are registered on clinicaltrials.gov investigating immunotherapy for liver cancer, with a majority of phase 2 studies. Endpoints may vary between overall survival (OS), progression-free survival (PFS), overall response rate, and disease control rate [1, 2]. Except OS, all endpoint surrogates are based on radiological size response and both the Food and Drug Administration and European Medicines Agency also accept a significant improvement of PFS as primary endpoint [3]. Conventional radiological responses (i.e. Response Evaluation Criteria in Solid Tumors, RECIST 1.1, or mRECIST, iRECIST, the European Association for the Study of Liver criteria [EASL]) are based on tumor-shrinking, which means decrease in size and/or decrease in enhancing tumor areas. However, an increase in PFS is not always associated with an increase in OS [4, 5]; and in the setting of immunotherapy and particularly of immune checkpoint inhibitors, conventional response criteria may not be reflective of tumor responses in liver cancer. Depending on which radiology criteria is used, assessment of tumor response can be different [6–10].

New patterns of response have been described, like pseudoprogression and dissociated response. Pseudoprogression describes a transitional growth in tumor diameter prior to response, which has been the main focus in the development of iRECIST. However, pseudoprogression is a rare phenomenon and is described in less than 5% of patients [11]. Dissociated responses is defined as mixed tumor response, where some target lesions decreased in size, whereas others have grown. This phenomenon might be more common, and has a reported frequency of 3.3–47.8% [12]. Using conventional response evaluation criteria, dissociated response will not be considered different than progressive disease, even if some studies showed prognosis might be better in that atypical response pattern [12–14].

Immunotherapy can also be combined with locoregional therapy which challenges the application of conventional response criteria even more [15–17]. Therefore, new quantitative imaging tools are needed to track complex tumor morphology evolution under treatment, to better predict tumor response and disease progression, perhaps taking into consideration specific modalities of therapy. Beyond size or volume, shape and margins are important features described by radiologist to differentiate malignant tumors from benign findings [18–20]. Shape descriptors, like solidity and circularity, can be used as objective quantitative features to evaluate the morphology of a tumor [21, 22]. Solidity is a quantitative measure of the shape irregularities and variation of the tumor borders, and does not refer to the necrotic liquid versus solid phase of the tumor [23]. Circularity is describing how close the shape is to a circle of the same area.

This study aimed to assess the feasibility of quantification of morphologic changes in tumors during immunotherapy, in patients who underwent combined subtotal locoregional treatment, as a reflection of response or survival, as an alternative, or as an addition to conventional imaging criteria.

## Methods and materials

### Population

A retrospective, single-center analysis was performed. All patients provided written informed consent prior to the study, and the study was approved by the National Cancer Institute (NCI) Institutional Review Board. In addition, a second IRB-approved protocol waived consent for image analysis and retrospective reporting. The study was conducted in conformity to the ethics guidelines of the 1990 Declaration of Helsinki and its amendments. Patients eligible were previously enrolled in a clinical trial (NCT01853618) and were treated by combined immunotherapy (tremelimumab ± durvalumab) and subtotal locoregional treatment (either thermal ablation or transarterial chemoembolization, with uncomplete coverage of the tumoral burden), between July 2013 and September 2020. Patients were all at least 18 years and had histopathological confirmation of primary liver cancer, and a disease not amenable to potentially curative liver transplantation, resection, or ablation. Exclusion criteria were (1) patients without measurable targets separate from targets treated by locoregional therapies, to avoid bias associated with variabilities in locoregional technique; (2) patients without follow-up CT or MRI available. 52 out of the 68 eligible patients have been reported [15, 16]. The prior articles dealt with safety and feasibility of combining immunotherapy with ablation in hepatocellular carcinoma [16] and refractory biliary tract cancer[15], whereas this work focuses on quantification of tumor morphological changes under treatment.

### Imaging

All patients underwent imaging studies at baseline (28 days maximum before or the first day of immunotherapy treatment) and then every 8 weeks. Imaging studies were preferentially multiphase contrast-enhanced CT using a 128 or 384-slices CT scanner (Somatom Definition Flash or Somatom Force, Siemens Healthineers, Erlanger, Germany). Images were acquired before and after injection of the contrast agent (Isovue-300, Milan, Italy) at arterial and portal venous phases. The imaging parameters were as follows: tube voltage, 120 kV; spiral pitch factor, 1. Images were reconstructed at a slice thickness of 2 mm, with a matrix of 512 × 512. In case of renal failure, abdominal MRI were performed on a 1.5 Tesla magnet (Aera, Siemens Healthineers, Erlanger, Germany). The MRI protocol, focusing on the upper abdominal region, included the following sequences: T1-weighted at in and out of phase echo times, T2-weighted turbo spin-echo, acquired in axial and coronal planes; diffusion-weighted, pre- and post-contrast (Gadavist, Bayer HealthCare Pharmaceuticals, Berlin, Germany) T1-weighted 3D gradient-echo sequences, acquired 20 s after injection (arterial) and 70 s after injection (portal venous), slice thickness:7.8 mm, matrix of 270 × 320.

### Conventional response evaluation

Conventional response (RECIST 1.1) and iRECIST were assessed at 6-month follow-up by one radiologist, blinded to clinical outcomes. Tumors treated with locoregional treatment were considered as non-measurable lesions for RECIST 1.1[24] and iRECIST evaluations. Patient with controlled disease included patients with either partial response or stable disease according to RECIST 1.1. Conventional response was compared to a second RECIST 1.1 evaluation from clinical trial data, using Kappa’s Cohen.

### Shape features analysis

The convex hull is the smallest convex polygon enclosing a planar tumor region of interest. The ratio between the area of the tumor mask and its convex hull quantifies substantial protuberances and depressions along the tumor border. As the value approaches 1.0, the shape is more regular and without concavity, whereas a lower solidity is associated with more irregular shape. Circularity is a 2D shape feature describing how close the shape is to a circle of the same area. A tumor with a circularity of 1.0 indicating a perfect circle. As the value approaches 0.0, it indicates an increasingly elongated shape.

The largest target lesion from RECIST 1.1 analysis was manually segmented on the axial slice with maximum diameter on contrast-enhanced CT or MRI scans. Shape descriptors including solidity (area/convex hull area) and circularity $\left( {4\pi \times {\text{Area}}} \right){\text{/Perimeter}}^{2}$ of tumor segmentation were calculated at baseline, 3-month follow-up and 6-month follow-up, using ImageJ software. Shape variation was defined as shape index at baseline—shape index at 6-month follow-up.

### Statistics

Normality of data was tested using the Shapiro–Wilk test. Kendall’s Tau test was used to assess the correlation between RECIST1.1 status and shape index or shape variations. The Friedman test was used to assess variability of shape over time. Kaplan Meier and Log-rank tests were performed for survival analyses. The optimal cut-off values of shape index and shape variations regarding survival stratification were determined using maximally selected rank statistics (“survminer” R package). Results were presented as median (interquartile range, IQR) or 95% confidence interval (CI). Statistics were performed using Rstudio (v2023.06.1 + 524).

## Results

### Patients

**Fig. 1:**
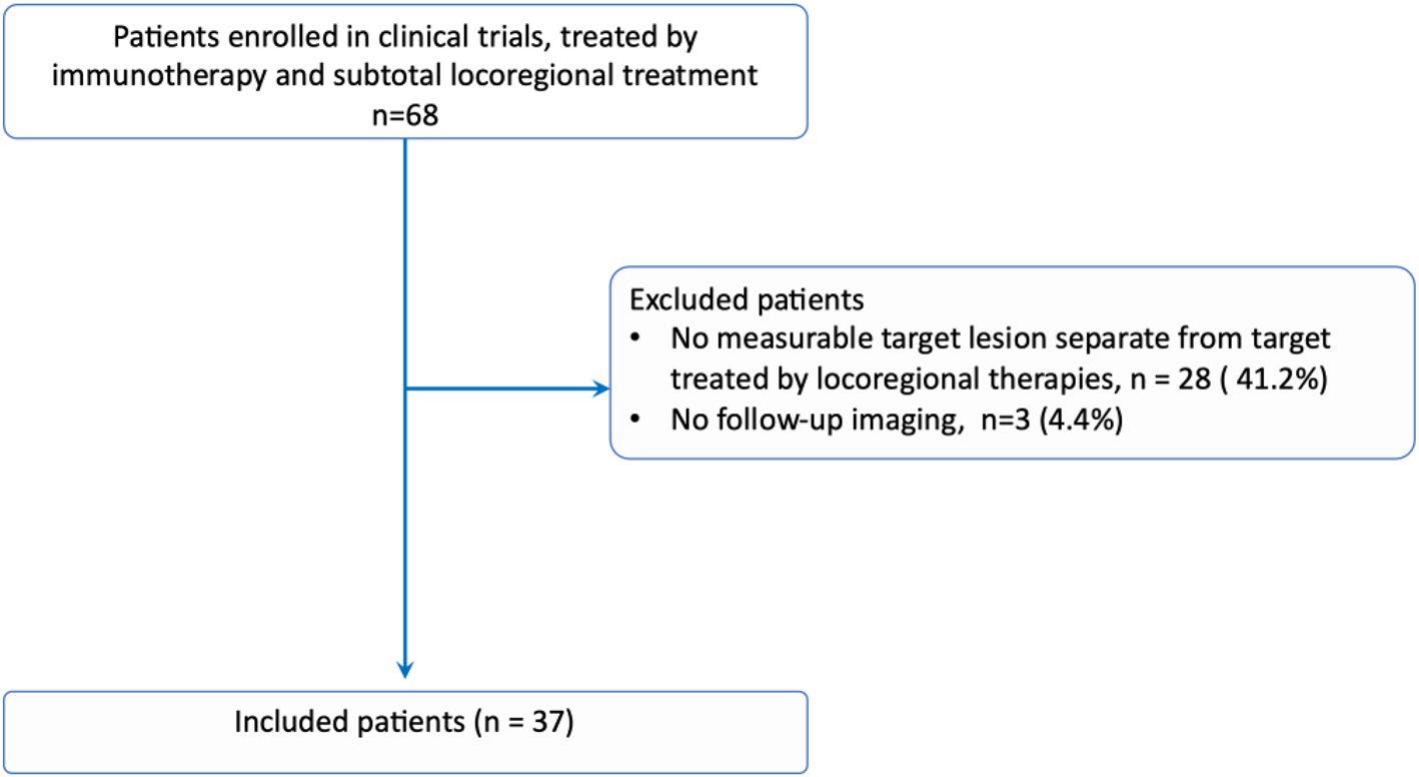
Study flowchart

From the 68 patients enrolled in the clinical trial, 28 (41.2%) did not have target lesions separate from lesions treated by locoregional therapies and 3 (4.4%) had no follow-up CT or MRI available. A total of 37 patients were included (7 females, 30 males). A flowchart of the study is presented in Fig. 1. 28/37 (75.7%) patients were diagnosed with hepatocellular carcinoma and 9/37 (24.3%) patients with biliary cancer (8 cholangiocarcinoma, 1 ampullary carcinoma). The median age was 64 years (IQR 56–68). 22/37 (59.5%) patients had extrahepatic disease. All patients received tremelimumab, and 6/37 (16.2%) received durvalumab. Locoregional treatment included cryoablations (7/37, 18.9%), microwave ablations (10/37, 27%), radiofrequency ablations (7/37, 18.9%), transarterial chemoembolization (11/37, 29.7%), and combinations of ablation and transarterial chemoembolization (2/37, 5.4%).

34/37 (91.9%) patients underwent multiphase CT scans with contrast injection. 3/37 (8.1%) patients had renal insufficiency or failure and underwent enhanced MRI. Tumors were segmented in the phase in which they were subjectively perceived to be most visible by the reviewing radiologist. For most of the patients (30/37, 81.1%), tumors were segmented on portal-phase imaging, but for 7/37 (18.9%) patients, tumors were segmented on arterial phase images.

All patients together, median PFS according to RECIST 1.1 was 147 days (IQR 98–230). In the subgroup of patients with HCC, median PFS was 238 days (IQR 142–314 days). In the subgroup of patients with biliary cancer, median PFS was 100 days (IQR 84-NA). All patients together, median OS was 301 days (IQR 221–525). In the subgroup of patients with HCC, median OS was 329 days (IQR 286–561). In the subgroup of patients with biliary cancer, median survival was 231 days (IQR 183-NA). The patient characteristics are presented in Table 1. Out of the 37 patients, 3 patients did not have a 6-month follow-up CT (2 had died, 1 was out of the study and did not have an imaging).

**Table 1:** Patient characteristics

Patient characteristics (*n* = 37)	
Age, years (interquartile range)	64 (56–68)
*Sex*	
Male	30 (81.1%)
Female	7 (18.9%)
*Pathology*	
Hepatocellular carcinoma	28 (75.7%)
Biliary cancer	9 (24.3%)
*Extrahepatic disease*	
Yes	22 (59.5%)
No	15 (40.5%)
*Immunotherapy Protocol*	
Tremelimumab	31 (83.7%)
Tremelimumab + Durvalumab	6 (16.2%)
Locoregional treatment	7 (18.9%)
Cryoablation	10 (27%)
Microwave ablation	7 (18.9%)
Radiofrequency ablation	11 (29.7%)
Transarterial chemoembolization	2 (5.4%)
Combination of locoregional treatment	
*Progression Free Survival, days (interquartile range)*	
Hepatocellular carcinoma	238 (142–314)
Biliary cancer	100 (84-NA)
All patients	147 (98–230)
*Overall survival, days (interquartile range)*	
Hepatocellular carcinoma	329 (286–561)
Biliary cancer	231 (183–NA)
All patients	301 (221–525)

### Conventional response evaluation

At the 6-month follow-up, according to RECIST 1.1, 18/34 (52.9%) patients had a progressive disease, 15/34 (44.1%) had a stable disease and 1/34 (2.9%) patient had a partial response. According to iRECIST, 13/34 (38.2%) had progressive disease, 5/34 (1.5%) had unconfirmed progressive disease, 15/34 (44.1%) had stable disease and 1/34 (2.9%) had a partial response. Disease control at 6-month follow-up according to RECIST 1.1 was associated with better survival (*p* = 0.01). Inter-reader agreement with a second reading for RECIST 1.1 was considered substantial (Cohen’s Kappa = 0.61).

### Shape analysis

**Fig. 2:**
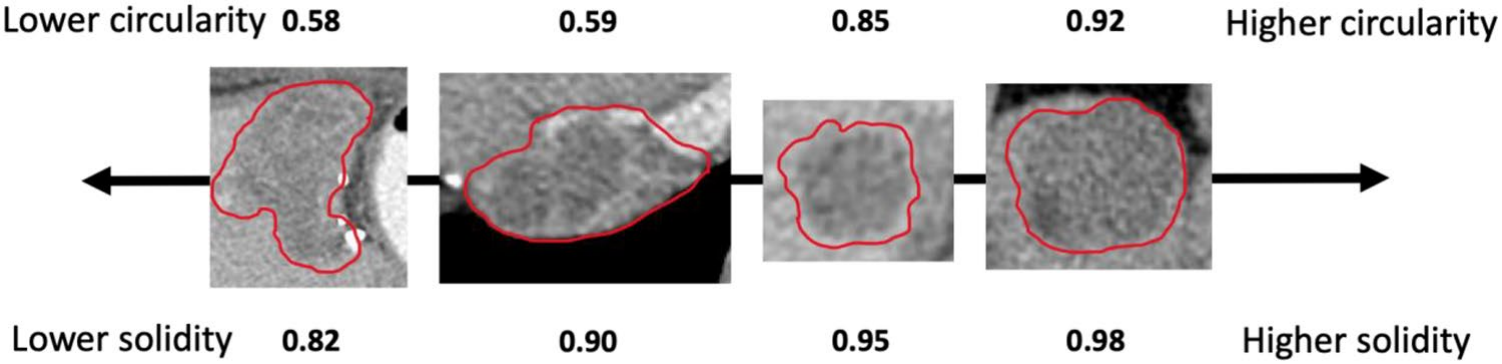
Example tumors with varying degrees of circularity and solidity. Tumor shapes were manually segmented on axial CT images (red line). Circularity $\left( {4\pi \times {\text{Area}}} \right){\text{/Perimeter}}^{2}$) and solidity (area/convex hull area of tumor) were calculated using ImageJ

**Fig. 3:**
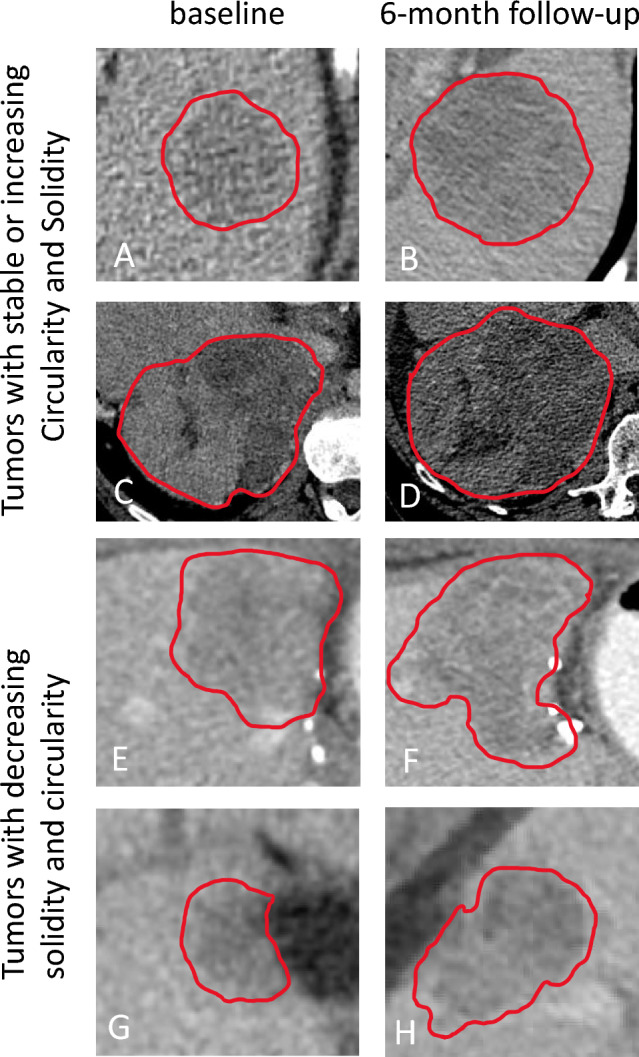
Four representatives cases showing the evolution of tumor shape between baseline (left) and 6-month follow-up (right). Tumors were manually segmented on axial CT images (red line). The two upper cases, (**A**–**D**) are tumors with stable or increasing solidity and circularity. The two lower cases, (**E**–**H**) are tumors with decreasing solidity and circularity (becoming more irregular and less circular tumors). Hepatocellular carcinoma (**A**, **B**) solidity: 0.96 then 0.97, and circularity 0.88 then 0.86. Adrenal metastasis of a hepatocellular carcinoma (**C**, **D**) solidity: 0.98 then 0.99 and circularity: 0.86 then 0.93. Cholangiocarcinoma (**E**, **F**) solidity: 0.95 then 0.82 and circularity: 0.76 then 0.58. Cholangiocarcinoma (**G**, **H**) solidity: 0.93 then 0.91 and circularity 0.80 then 0.64.

Segmented target tumors at baseline included liver tumors (25/37, 67.6%), lymphadenopathy (7/37, 18.9%), adrenal metastasis (1/37, 2.7%), lung metastasis (1/37, 2.7%), peritoneal nodule (1/37, 2.7%), spleen metastasis (1/37, 2.7%) and bone metastasis (1/37, 2.7%). One hundred one tumor segmentations were analyzed (37 at baseline, 31 at 3-month follow-up and 33 at 6-month follow-up). Figure 2 presents examples of tumor shapes, with different circularity and solidity. Median circularity was 0.835 (IQR 0.751–0.877) at baseline, 0.828 (IQR 0.775–0.867) at 3-month follow-up and 0.815 (IQR 0.697–0.853) at 6-month follow-up. Median solidity was 0.958 (IQR 0.936–0.969) at baseline, 0.957 (IQR 0.943–0.969) at 3-month follow-up and 0.955 (IQR 0.918–0.969) at 6-month follow-up. Circularity changed over time (*p* = 0.04), but there was no difference in solidity over time (*p* = 0.16). Figure 3 presents four representative cases of shape evolution from baseline to 6-month follow-up.

### Shape variation according to RECIST 1.1

**Fig. 4:**
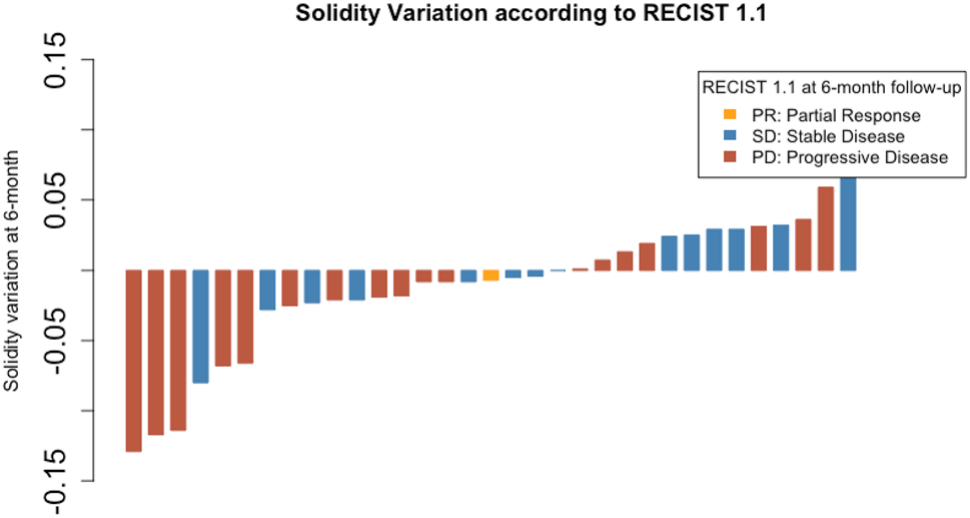
Waterfall plot for target lesion solidity variation according to RECIST 1.1 status at 6-month follow-up. No correlation between circularity variation and RECIST 1.1 status was found

No correlation was found between RECIST 1.1 status at 6-month follow-up and circularity variation (*p* = 0.27) or solidity variation (*p* = 0.25), or between RECIST 1.1 status at 6-month follow-up and circularity (*p* = 0.15) or solidity (*p* = 0.07) at 6-month follow-up. Waterfall plot for target solidity variations, according to RECIST 1.1 status at 6-month follow-up is presented in Fig. 4. No correlation was found between size variation at 6-month follow-up and circularity variation (*p* = 0.44) or solidity variation (*p* = 0.30); or between size variation at 6-month follow-up and circularity (*p* = 0.84) or solidity (*p* = 0.57) at 6-month follow -up.

### Survival analysis

**Fig. 5:**
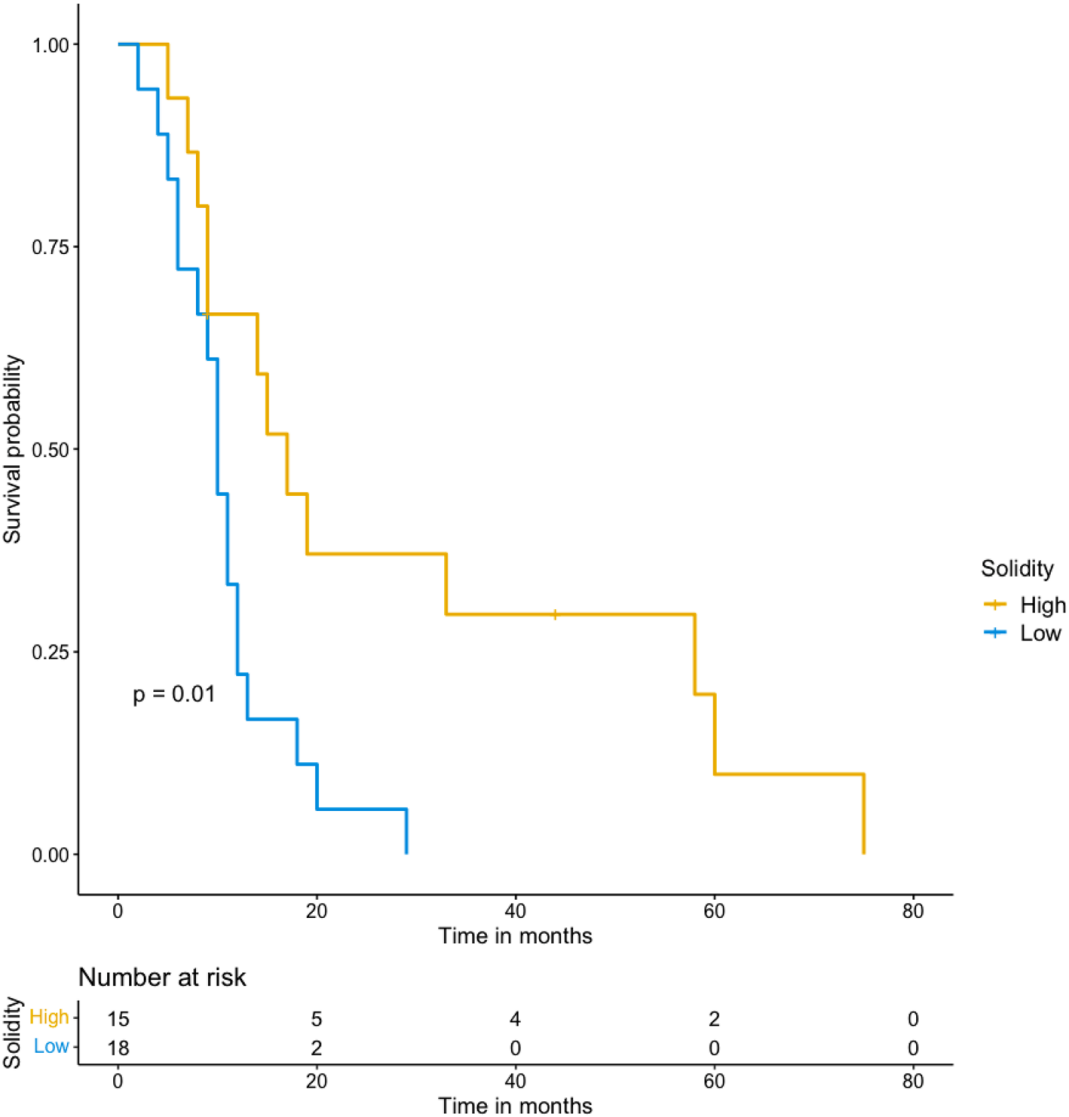
Survival analysis according to solidity of target at 6-month follow-up

Patients with low solidity tumors at 6-month follow-up showed poorer prognosis compared with patients with high solidity tumors at 6-month follow-up (*p* = 0.01). Figure 5 illustrates the survival analysis according to solidity at 6-month follow-up. The optimal threshold for solidity according to maximally selected rank statistics at 6-month follow-up was a solidity of < 0.956. Median survival was 304 days (95% CI 230–403) for patients with low solidity tumors at 6-month follow-up and 525 days (95% CI 269-NA) for patients with high solidity tumors at 6-month follow-up.

**Fig. 6:**
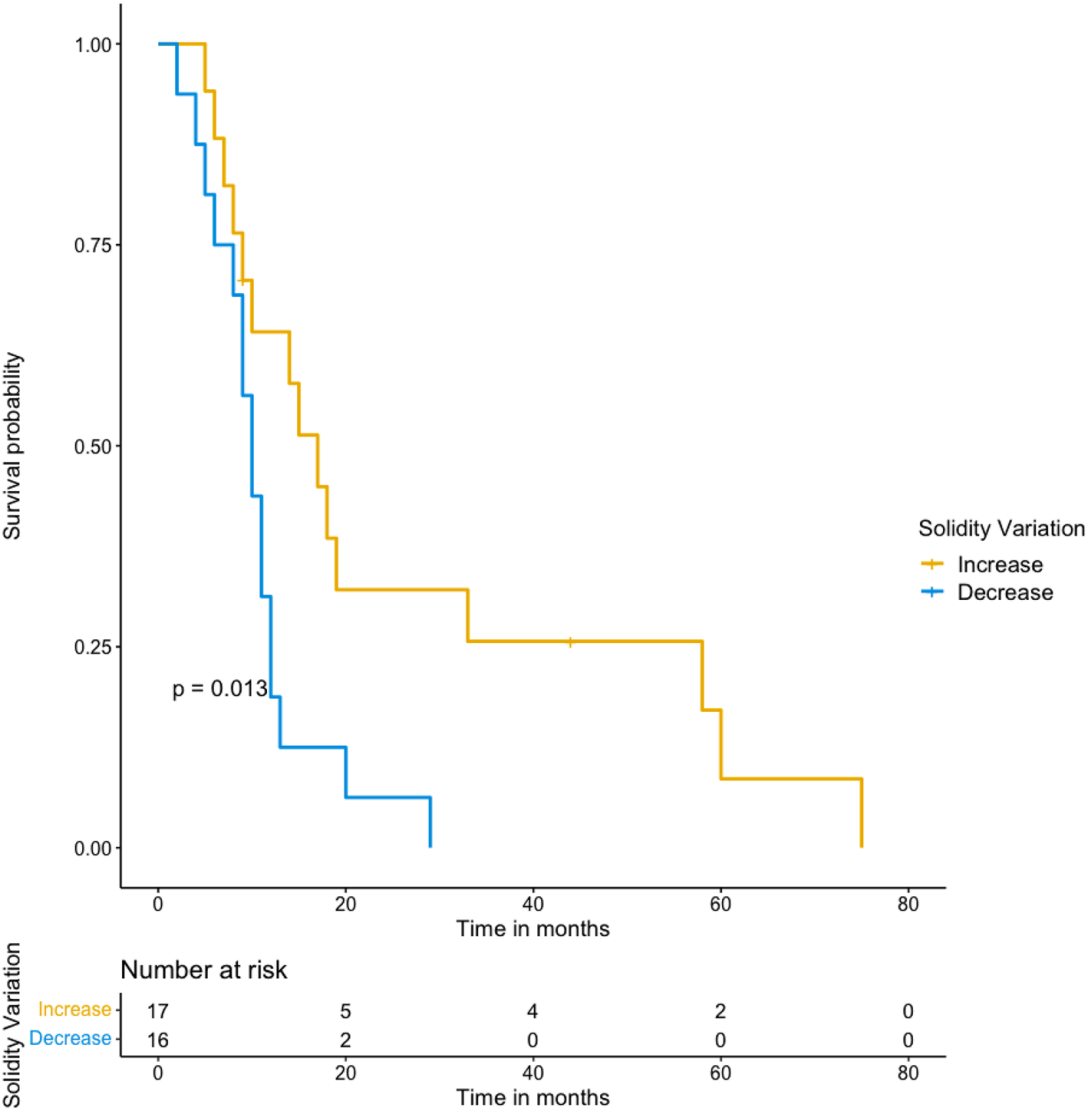
Survival analysis according to target solidity variation between baseline and 6-month follow-up. Patient with tumor becoming more irregular, or having a decreasing solidity, between baseline and 6-month follow-up, showed poorer prognosis than patients with tumor becoming more regular, or having an increasing solidity (*p* = 0.01)

Solidity variation analysis confirmed that a decrease of tumor solidity (increasing irregularity) at 6-month follow-up was associated with poorer prognosis (*p* = 0.01). Figure 6 shows Kaplan–Meier curves according to solidity variation at 6-month follow-up.

No association was found between shape features at baseline and overall survival, or between shape features at 3-month follow-up and overall survival (solidity, *p* = 0.3; circularity, *p* = 0.15). No association was found between circularity at 6-month follow-up and overall survival (*p* = 0.18).

Patients with irregular tumor, or low solidity, at 6-month follow-up have poorer prognosis than patients with more regular tumor, or higher solidity at 6-month follow-up (*p* = 0.01).

## Discussion

Tumor morphology can evolve during treatment and disease progression, and it is possible to quantify some changes using shape descriptors. In this study, we showed that it is feasible to measure the variations of solidity and circularity of a tumor, and that some of those variations may independently correlate with survival in patients with liver cancer treated by immunotherapy combined with subtotal locoregional treatment (ablation or embolization).

Previous publications have described how shape of tumors could provide information about prognosis or predict response to treatment. Shape analysis has been shown to correlate with histopathology in prostate cancer [20]. Solidity was associated with overall survival in pre-operative lung adenocarcinoma [25]. The analog of circularity in 3 dimensions, sphericity, was associated with tumor response in nasopharyngeal carcinoma after intensity modulation radiation therapy [26]. In this study, circularity and solidity variation did not show a correlation with RECIST 1.1 status at 6-month follow-up, suggesting that circularity and solidity variation may provide additional information about tumor evolution in patients undergoing immunotherapy. In this study, progressive disease according to RECIST 1.1 at 6-month follow-up was associated with a poorer prognosis. Both RECIST 1.1 and solidity variation could therefore provide complementary and augmentative information about disease progression. The combination of conventional size criteria and shape criteria was not evaluated in this small study but would be best evaluated in larger studies.

This study showed that patient with low solidity tumor at 6-month follow-up as well as a decrease of solidity at 6-month follow-up were associated with poorer prognosis. Decrease of solidity means a more irregular tumor which is consistent with a malignant tumor at diagnosis for breast [18] and lung [19] findings. Although manual segmentation of each target tumor is not practical in clinical practice, the awareness that shape may also matter in addition to size or largest diameter. Thus changes in tumor morphology might also give relevant information about prognosis and possible benefit of the immunotherapy. Whether shape might relate to underlying genotype or specific molecular pathways remains unknown.

This study has several limitations. First, segmentations were performed in 2 dimensions in the maximal axis slice. Even if 3D volume segmentation might have given more detailed information, 2D manual segmentation may be more easily translated into clinical practice. Volume measurements have been shown to be more strongly correlated to prognosis than 2 dimensions maximal axis [27, 28], however 2D maximal axis measurements are still used [29] as it is less time consuming. This study also included a heterogeneous group, in terms of pathology, disease stage, prior treatment, and ongoing treatments, from a single center. Moreover, shape analysis by organ of interest and by tumor type (primary tumor versus metastasis) was not feasible because of the small cohorts with limited numbers of patients.

Further studies are needed to confirm these findings. Deep-learning segmentation tools could enable automatic segmentation and three-dimensional analyses. This study compared shape features to RECIST, considered the gold standard for oncological patients, however, more morphologic or imaging features such as enhancement and wash-out could be added in the analysis using radiomics tools.

## Conclusion

Tumor morphology can evolve during treatment and disease progression. Shape descriptors can be used to quantify those changes. A tumor becoming more irregular at 6-month follow-up, or decreasing in its solidity, may correlate with poorer prognosis, in patients with liver cancer treated by immunotherapy and subtotal locoregional treatment. Future consideration should be given towards integrating shape features into immunotherapy response criteria, given that shape feature analysis itself may correlate with response. Conventional size criteria augmented by shape features may enhance performance of response criteria in terms of prediction of outcomes and survival.
